# Repurposing metformin as a quorum sensing inhibitor in *Pseudomonas aeruginosa*

**DOI:** 10.4314/ahs.v17i3.24

**Published:** 2017-09

**Authors:** Hisham A Abbas, Ahmed M Elsherbini, Moutaz A Shaldam

**Affiliations:** 1 Department of Microbiology and Immunology-Faculty of Pharmacy-Zagazig University- Zagazig- Egypt; 2 Health Sciences College-Umm Al Qura University, AlQunfudah, Saudi Arabia; 3 Department of Medicinal Chemistry, Faculty of Pharmacy, Delta University for Science and Technology, Gamasa, Egypt

**Keywords:** Metformin, *Pseudomonas aeruginosa*, quorum sensing, virulence inhibition

## Abstract

**Background:**

Quorum sensing is a mechanism of intercellular communication that controls the production of virulence factors in *Pseudomonas aeruginosa*. Inhibition of quorum sensing can disarm the virulence factors without exerting stress on bacterial growth that leads to emergence of antibiotic resistance.

**Objectives:**

Finding a new quorum sensing inhibitor and determining its inhibitory activities against virulence factors of *Pseudomonas aeruginosa* PAO1 strain.

**Methods:**

Quorum sensing was evaluated by estimation of violacein production by *Chromobacterium violaceum* CV026. Molecular docking was used to investigate the possible binding of metformin to LasR and rhlR receptors. The inhibition of pyocyanin, hemolysin, protease, elastase in addition to swimming and twitching motilities, biofilm formation and resistance to oxidative stress by metformin was also assessed.

**Results:**

Metformin significantly reduced the production of violacein pigment. Significant inhibition of pyocyanin, hemolysin, protease and elastase was achieved. Metformin markedly decreased biofilm formation, swimming and twitching motilities and increased the sensitivity to oxidative stress. In the molecular docking study, metformin could bind to LasR by hydrogen bonding and electrostatic interaction and to rhlR by hydrogen bonding only.

**Conclusion:**

Metformin can act as a quorum sensing inhibitor and virulence inhibiting agent that may be useful in the treatment of *Pseudomonas aeruginosa* infection.

## Introduction

*Pseudomonas aeruginosa* is an opportunistic pathogen that affects patients with suppressed immunity. It is the etiologic agent of a number of nosocomial infections including pneumonia, burns, wounds, diabetic foot and urinary tract infections^1^–^3^.

Antibiotics are considered the first choice in treating bacterial infections. However, the resistance of bacteria to antibiotics is out pacing the poor supply of new antibiotics. As a result, it is extremely necessary to develop new therapeutic approaches to overcome antibiotic resistance^4^. One of these promising approaches is the interference with the virulence of bacteria. This can be advantageous due to the importance of virulence factors in pathogenesis. Moreover, targeting virulence makes no stress on bacterial growth and can avoid the emergence of resistance^5^,^6^.

Quorum sensing is involved in regulation of virulence of bacteria. Quorum sensing is a type of intercellular signaling in bacteria. This can take place by secretion of signaling molecules or auto-inducers by the bacterial cells. These auto-inducers are proportional in their concentration to the bacterial cell number. When reaching a critical concentration; the quorum, the activation of the genes encoding virulence factors occurs^7^. Quorum sensing controls of the production of *P. aeruginosa* virulence factors such as elastase, protease, hemolysin, pyocyanin, in addition to swimming and twitching motilities, biofilm formation and resistance to oxidative stress. There are three quorum sensing systems in *Pseudomonas aeruginosa*; the LasI/LasR system, the rhlI/rhlR system and the PQS/ MvfR system. The auto-inducer 3-oxodecanoyl homoserine lactone (C12 HSL) is produced by LasI and binds to the cognate receptor LasR, while rhlI controls the production of the butanoyl homoserine lactone (C4 HSL) that is recognized by rhlR receptor. The signaling molecule of the PQS-MvfR system is 2-heptyl-3-hydroxy-4(1H) quinolone (PQS) and its cognate receptor is MvfR. PQS-MvfR system functions in regulation of the transcription of downstream targets^8^–^12^.

Metformin is one of the most commonly prescribed oral hypoglycemic for treatment of type 2 diabetes. It is a biguanide drug that is useful especially for obese patients^13^,^14^. Metformin has anti-bacterial activity against *Pseudomonas aeruginosa*^15^. Moreover, it was found to potentiate the antibacterial activities of gold nanoparticles against superbugs, probably due to the effect on the cell wall. Furthermore, it augmented the biofilm eradicating activity of gold nanoparticles^16^.

This study aimed to investigate the possible anti-quorum sensing and anti-virulence activities of metformin in *P. aeruginosa* PAO1 strain.

## Materials and methods

### Media and chemicals

Luria-Bertani (LB) broth, LB agar and tryptone were obtained from Lab M Limited (Lancashire, United Kingdom). Mueller Hinton broth, Mueller Hinton agar and Tryptone soya broth and Tryptone soya agar were the products of Oxoid (Hampshire, UK). Metformin hydrochloride, dimethyl sulphoxide (DMSO), N-hexanoyl homoserine lactone and elastin congo red were purchased from Sigma (St. Louis, USA). Other chemicals were of pharmaceutical grade.

### Bacterial strains

*Pseudomonas aeruginosa* PAO1 strain was kindly provided by the Department of Microbiology, Faculty of Pharmacy, Mansoura University, while *Chromobacterium violaceum* CV026 was kindly gifted by the Department of Microbiology, Faculty of Pharmacy, Ain Shams University.

### Determination of minimum inhibitory concentration (MIC) and the effect of sub-inhibitory concentration of metformin on bacterial growth

The minimum inhibitory concentration of metformin was determined by the broth microdilution method (CLSI)^17^. Two fold serial dilutions of metformin in Mueller-Hinton broth (200, 100, 50, 25, 12.5, 6.25, 3.125, 1.56 mg/ml) were added in 100 µl aliquots to the wells of 96-wells microtiter plate. To each dilution, 100 µl of PAO1 suspension in Mueller-Hinton broth with 1 x 10^6^ CFU/ml were added. After 20 h of incubation of the plate at 37°C, the minimum inhibitory concentration was determined as the lowest concentration that showed no visible growth.

The effect 1/10 MIC of metformin on growth of PAO1 was determined as modified from Nalca et al.^18^ LB broth was inoculated with overnight culture of PAO1 in the presence and absence of metformin and overnight incubated at 37 °C. The turbidities of untreated and metformin treated cultures were measured at 600 nm using Biotek Spectrofluorimeter (Biotek, USA).

### Violacein inhibition assay

The production of the quorum sensing violacein pigment was estimated according to Choo et al.^19^ Overnight culture of *Chromobacterium violaceum* CV026 was prepared and adjusted to an OD600 of 1. After addition of aliquots of 100 µl of LB broth containing N-hexanoyl homoserine lactone with and without 1/10 MIC of metformin to the wells of a 96-well microtiter plate, the bacterial suspension was added in aliquots of 100 µl. After 16 h of incubation at 28°C, the plate was completely dried at 60°C. To extract violacein, 100 µl of DMSO was delivered to each well followed by incubation at 30°C with shaking. Negative control using DMSO alone was prepared. Violacein was quantified by measuring the absorbance at 590 nm using Biotek Spectrofluorimeter (Biotek, USA).

### Assay of biofilm formation and inhibition

For assay of biofilm inhibition, biofilms were formed in the absence and presence of 1/10 MIC of metformin by using the modified method of Stepanovic et al.^20^ PAO1 was grown in TSB and diluted to approximate density of 1 × 10^6^ CFU/ml. The bacterial suspension (0.1 ml) was added to the wells of 96 well sterile microtiter plate with and without 1/10 MIC of metformin followed by incubation at 37°C for 24 h. The planktonic cells were discarded and the wells were washed 3 times with sterile phosphate buffered saline (PBS, pH 7.2). Methanol (99%) was added to the wells for 20 minutes as a fixing agent and crystal violet (1%) was used to stain the wells for 20 minutes. The stain was washed with distilled water, the plate was air-dried and 33% glacial acetic acid was used to solubilize crystal violet. The absorbance was measured with Biotek spectrofluorimeter (Biotek, USA) at 590 nm.

### Microscopic analysis of biofilm inhibition

*P. aeruginosa* PAO1 was grown overnight in TSB and its optical density was adjusted (OD600 = 1). Fresh media with sterilized cover slips was inoculated with the adjusted bacterial culture in 50 ml centrifuge tubes with and without 1/10 MIC of metformin. The tubes were incubated at 37°C for 16 h and the cover slips were washedwith phosphate-buffered saline. The attached biofilms were stained with crystal violet (1%) and examined under the light microscope using the oil immersion lens (100x magnification)^21^.

### Swimming and twitching motilities assay

The effect of metformin on swimming and twitching was detected according to the method of Rashid and Kornberg^22^ with some modifications. For swimming assay, swimming agar plates (tryptone1%, sodium chloride 0.5% and agar 0.3%) with and without metformin (1/10 MIC) were prepared. Overnight culture of PAO1 in tryptone broth was prepared, diluted, and 5µl were stabbed into the center of the agar plates and incubated for 24h at 37°C. The swimming zones were measured.

In twitching assay, 1% LB agar plates containing metformin (1/10 MIC) and control plates were stab-inoculated with 2µl of the prepared culture and incubated at 37°C for 48h. The agar was removed, the plates were air-dried and stained with crystal violet. The dye was removed and the plates were washed with water and dried. The twitching zones were measured.

### Protease assay

To estimate the protease inhibiting activity of metformin, the skim milk agar method was used according to Vijayaraghavan and Vincent^23^. *P. aeruginosa* PAO1 overnight cultures in LB broth with and without 1/10 MIC of metformin were prepared. The supernatants were separated by centrifugation at 10,000 rpm for 15 min. skim milk agar plates (5%) were prepared and supernatants were added (100 µl) to the wells made in the skim milk agar plates. The plates were incubated overnight at 37°C and the clear zones surrounding the wells were measured to determine the proteolytic activity in the presence and absence of metformin.

### Elasatse assay

The elastin congo red assay was used to estimate the elastolytic activity of untreated and metformin treated *Ps. aeruginosa* PAO1 cultures according to Ohman et al.^24^ Supernatants were prepared in the same way as in protease assay and added in amounts of 0.5 ml to tubes with elastin congo red reagent [10 mg of ECR and 0.5 ml of buffer composed of Tris pH 7.2 (0.1 mol/l) and CaCl^2^ (10 mol/l)]. After 6 h of incubation at 37°C with shaking, insoluble elastin congo red was discarded by centrifugation and the absorbance was measured at 495 nm using Biotek Spectrofluorimeter (Biotek, USA).

### Pyocyanin assay

The production of pyocyanin by untreated and metformin treated PAO1 was assayed by the method of Das and Manefield^25^. PAO1 overnight culture in LB broth was prepared and diluted to OD600 of 0.4. LB broth (1 ml) with and without metformin was inoculated with 10µl of the diluted suspension and the cultures were incubated at 37°C for 48h. To remove the cells and obtain the supernatants, the tubes were centrifuged at 10,000 rpm for 10 minutes and the pyocyanin was quantified in the supernatants by measuring the absorbance at 691nm by Biotek Spectrofluorimeter (Biotek, USA).

### Sensitivity to oxidative stress

The modified disk assay method of Hassett et al.^26^ was used to determine the ability of metformin to inhibit resistance to oxidative stress. Overnight cultures of PAO1 in LB were prepared and 0.1 ml aliquots were uniformly spread on the surface of LB agar plates containing 1/10 MIC of metformin. Ten µl of hydrogen peroxide (1.5%) was added to sterile paper disks (6 mm) present on the surface of LB agar plates. Control plates without metformin were prepared in the same way. The plates were incubated for 24 h at 37°C and the inhibition zones were measured.

### Docking study

To determine the molecular interaction of metformin with the LasR, rhlR receptors, docking study was performed. The crystal structure of *P. aeruginosa* LasR ligand binding domain was obtained from Protein Data Bank (PDB ID: 2UV0)^27^, while the rhlr receptor model (ID: P54292.1) was achieved from protein model portal28. The interaction of metformin, the natural ligand, 3-oxo-dodecanoylhomoserine lactone and the classical quorum sensing inhibitor C30 furanone into the receptor active site was investigated using Molegro Virtual Docker (MVD Version 6.0). Metformin was drawn into Marvin Sketch V5.11.5.29, and, for docking, and the most energetically favored conformer was saved in the file format of (*.mol^2^). The optimal geometry of the ligand was determined during the docking process and the E monomer was selected for analysis. Water was discarded, cavities were determined and the search area was set to be 9 Å from the center of the active site. Iterative simplex algorithm was selected to perform docking process with 10 runs per ligand, 200 population size, 1000 max iteration and 5 poses for each ligand. MolDock docking engine^30^ employing docking template and the optimized ligands was executed. Finally, the top returned poses were selected for analysis. Similarly, docking metformin and the autoinducer, butanoyl homoserine lactone (C4-HSL), into the active site of the rhlr receptor model was performed.

### Statistical analysis

Student t tests, Graph Pad Prism 5 was used to investigate the significance of the inhibitory activities of metformin against protease, swimming and twitching motilities, biofilm formation and eradication, violacein, pyocyanin, elastase oxidative stress, and hemolysin. P values <0.05 were considered statistically significant.

## Results

### Antibacterial activity of metformin against *Pseudomonas aeruginosa* PAO1

Metformin inhibited the growth of *Pseudomonas aeruginosa* PAO1 at 100 mg/ml. The activity of metformin against quorum sensing and virulence was evaluated at 1/10 MIC (10 mg/ml).

### Growth inhibition assay

**Figure 1:**
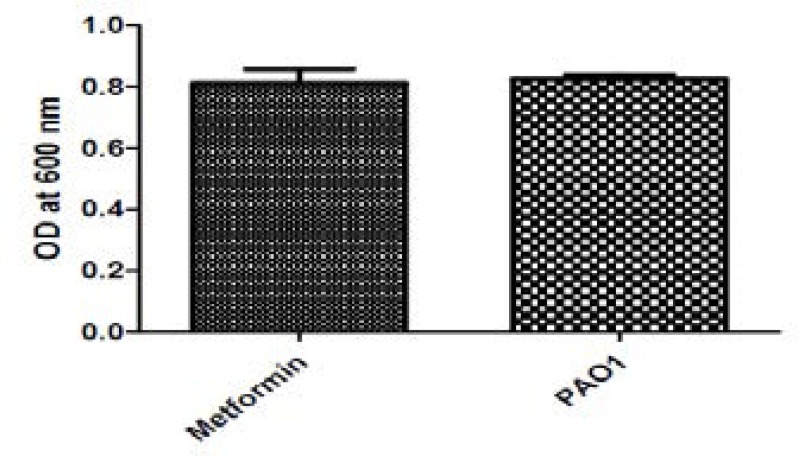
Effect of metformin on growth of PAO1. The growth was measured by measuring OD600 after overnight incubation in the absence and presence of 1/10 MIC of metformin. No statistically significant difference was found between the growth rates of the untreated or treated cultures.

The effect of metformin on QS and virulence may be due to the effect on growth of PAO1. To exclude this possibility, the effect of 1/10 MIC of metformin on growth was investigated by measuring the absorbance of the bacterial suspensions at 600nm after overnight incubation in LB broth.There was no statistically significant difference in the growth rate in the presence or absence of metformin (Fig. 1).

### Biofilm inhibition activities of metformin

**Figure 2:**
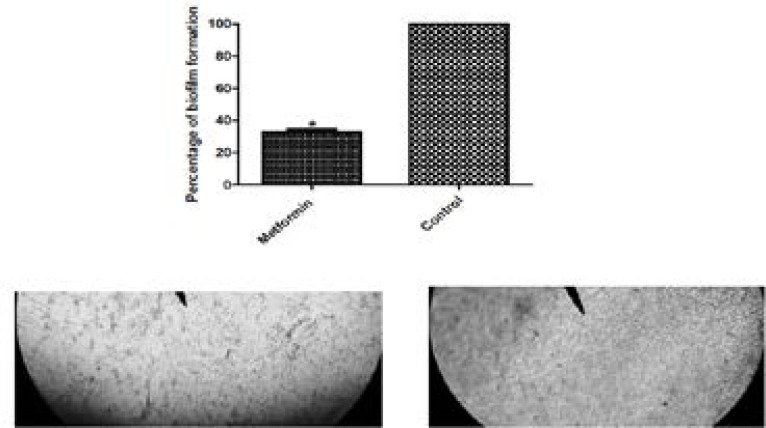
Inhibition of biofilm formation of PAO1 by 1/10 MIC of metformin.*, significant P< 0.05.Microscopic visualization of biofilm under the light microscope (X1000) in the treated culture (left) and untreated (right).

Metformin caused a significant decrease in biofilm formation. It could inhibit biofilm formed by PAO1 strain by 67.9% (Fig. 2). To visualize the inhibitory activity of metformin on biofilm formation, biofilms were formed on glass sterile cover slips in the absence and presence of metformin and examined after staining with crystal violet under the light microscope. As compared to the control, the cells in treated sample show scattered appearance.

### Inhibition of violacein production

**Figure 3:**
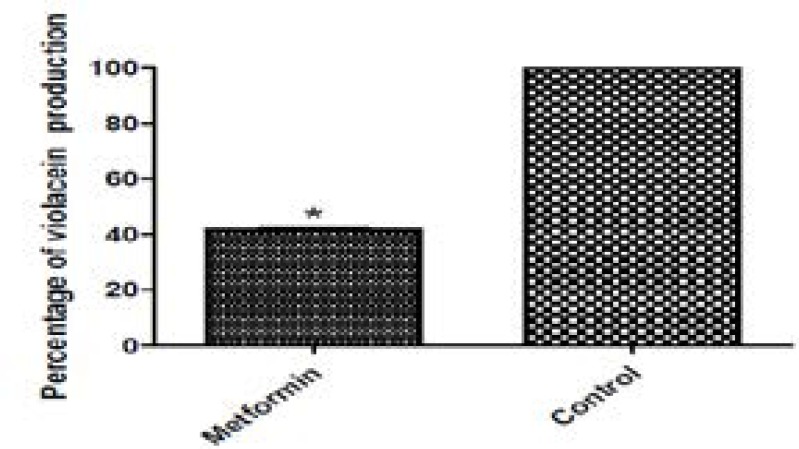
Inhibition of production violacein pigment of ChhromobacteriumviolaceumCV026 by 1/10 MIC of metformin.*, significant P< 0.05.

Quorum sensing was evaluated quantitatively by measuring the production of the purple pigment violacein in the biosensor strain *C. violaceum* CV026. This mutant can produce the pigment only when acylhomoserine lactone is added to the growth media. Sub-inhibitory concentration of metformin reduced violacein production in *C. violaceum* CV026 by 58.48% (Fig. 3)

### Protease inhibition assay

**Figure 4:**
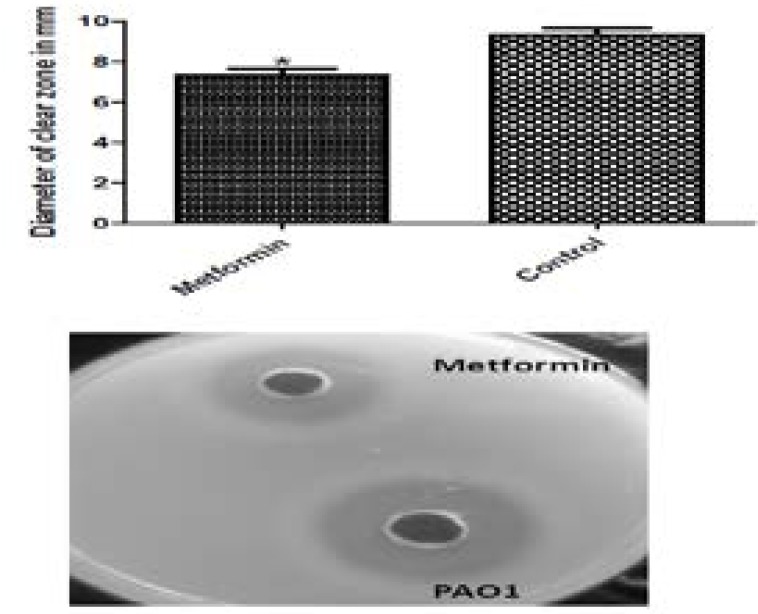
Inhibition of protease production by 1/10 MIC of metformin by the skim milk agar method. Metformin reduced the diameter of clear zone around the wells made in skim milk agar. *, significant P< 0.05.

The ability of metformin to reduce protease production by *Pseudomonas aeruginosa* PAO1 strain was evaluated by measuring the clearance zone surrounding the wells in skim milk agar after adding the supernatant of the treated and untreated cultures. Metformin was capable of reducing the proteolytic activity by 21.48% (Fig. 4).

### Pyocyanin inhibition assay

**Figure 5:**
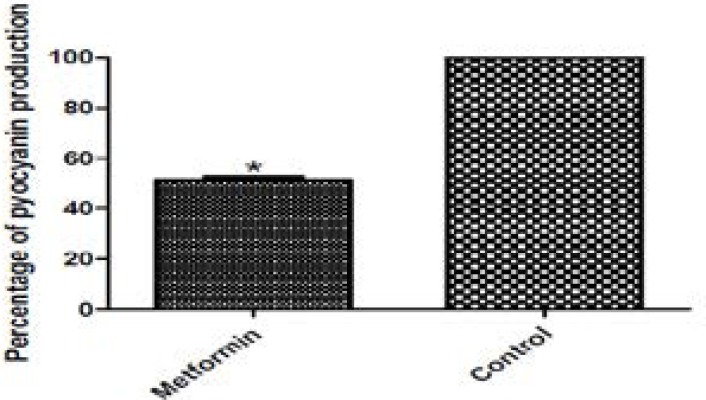
Pyocyanin production in PAO1 untreated (left) and metformin treated (right). The supernatants of both treated and untreated cultures were measured at 691nm. Metformin significantly reduced pyocyanin production. *, significant P< 0.05.

Pyocyanin is the green pigment that acts as a redox metabolite in *P. aeruginosa*. It is cytotoxic to lung tissue during infection^31^. The pyocyanin production in PAO1 was assayed in the absence and presence of metformin. Metformin showed good inhibiting activity against pyocyanin production. It reduced its production by 48.67% (Fig 5).

### Elastase inhibition assay

**Figure 6:**
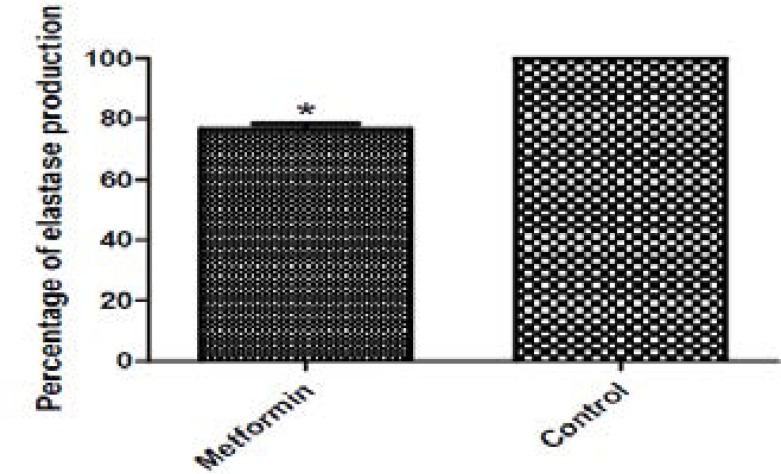
Inhibition of elastase activity by 1/10 MIC of metformin. The supernatants of untreated and metformin treated cultures were incubated with elastin congo red. After centrifugation to remove the insoluble dye, the supernatants were measured at 495nm. Significant decrease in elastase activity was found with metformin. *, significant P< 0.05.

The elastolytic activity of untreated PAO1 culture supernatant in addition to the metformin treated one was measured by the investigating ability of elastase enzyme to degrade elastin congo red. The production of elastase in the presence of metformin was diminished as related to the control. The percentage of inhibition was in the range of 23. 26% (Fig. 6).

### Oxidative stress assay

**Figure 7:**
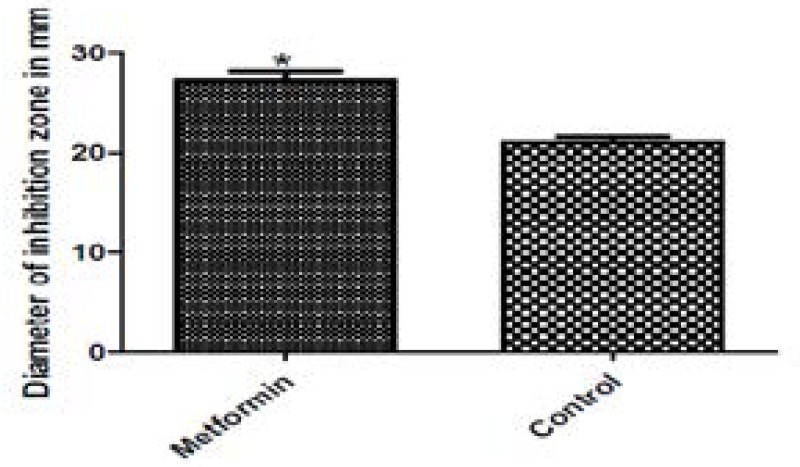
Effect of metformin on resistance to oxidative stress in PAO1.The diameter of inhibition zone of hydrogen peroxide in the presence of metformin (left) and in absence of metformin (right).*, significant P< 0.05.

The tolerance to oxidative stress was evaluated by testing the inhibitory effect of hydrogen peroxide on the growth of PAO1 in the presence and absence of metformin. Metformin could augment the effect of hydrogen peroxide or reduce the tolerance to oxidative stress by 23.15% (Fig. 7).

### Inhibition of swimming and twitching motilities

**Figure 8:**
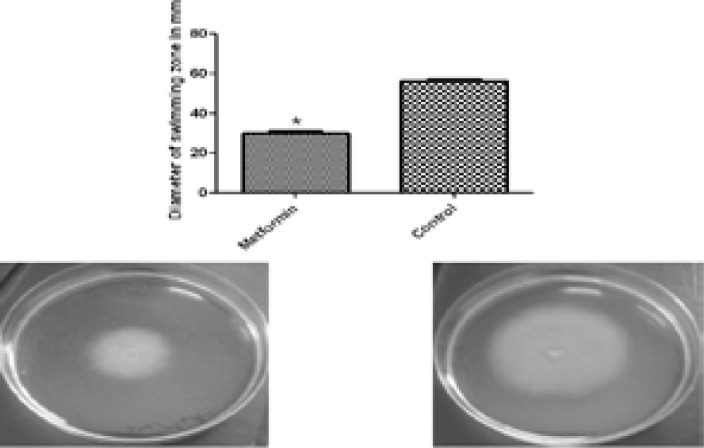
Inhibition of swimming motility of PAO1 by 1/10 MIC of metformin. Metformin decreased swimming (left) as compared to the control (right). *, significant P< 0.05.

**Figure 9:**
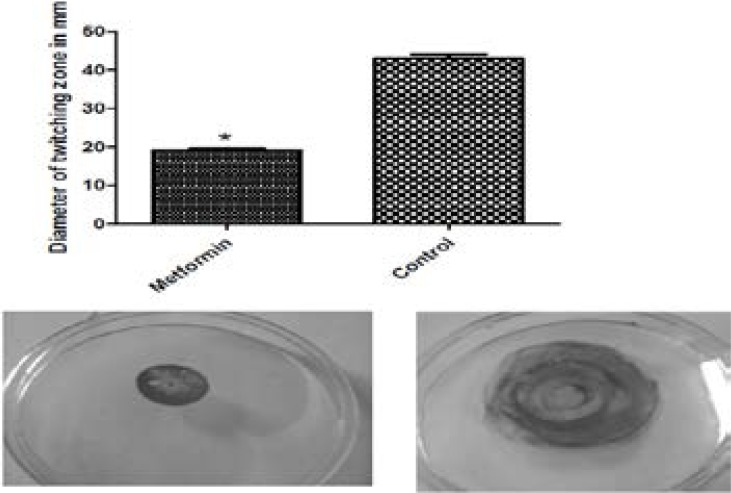
Inhibition of twitching motility of PAO1 by 1/10 MIC of metformin. Metformin decreased twitching (left) as compared to the control (right). *, significant P< 0.05.

The assay of the activity of metformin against swimming and twitching motilities was performed and metformin was able to inhibit swimming motility by 46.78% (Fig. 8) and twitching motility by 55.82% (Fig. 9).

### Inhibition of hemolytic activity

**Figure 10:**
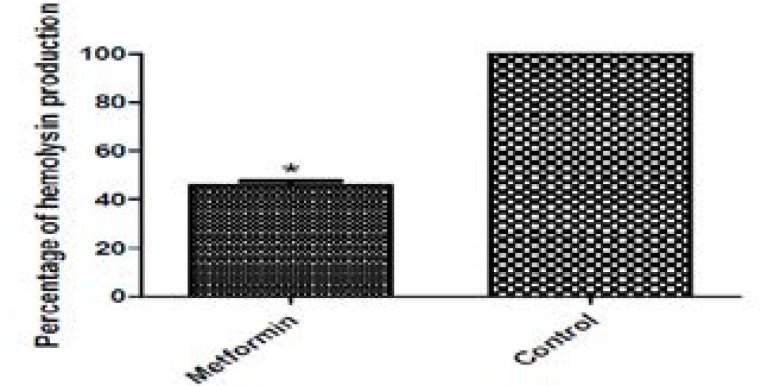
Inhibition of hemolysin by metformin. Hemoglobin was released by incubation of culture supernatants with 2% erythrocyte suspension in saline for 2h and the absorbance was measured at 540nm. Metformin showed a significant hemolysin inhibiting activity. *, significant P< 0.05.

The hemolytic activity of untreated PAO1 and metformin treated culture supernatants was assayed quantitatively and the hemoglobin release was measured spectrophotometrically. Metformin could inhibit hemolytic activity of PAO1 by 54.27% as compared to the untreated control (Fig. 10).

### Docking study

**Figure 11:**
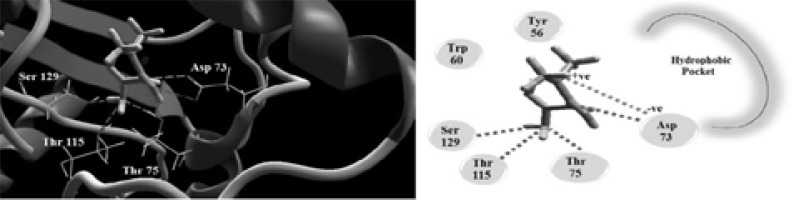
The Molecular docking of metformin into the active site of LasR enzyme 3D (Left) and 2D schematic view of the binding (Right).

**Figure 12:**
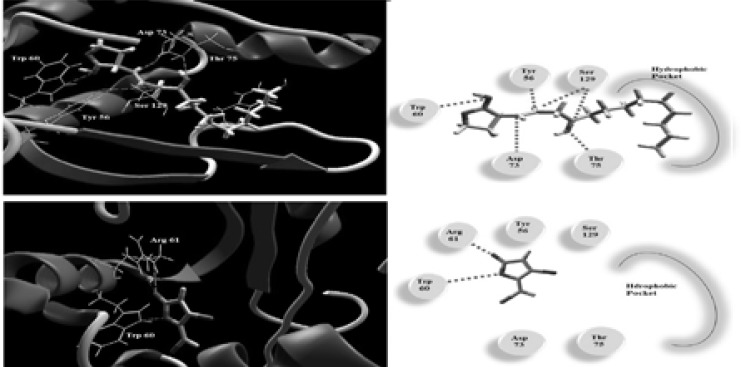
The Molecular docking of natural ligand (Top) and C30 furanone (Bottom) into the active site of LasR enzyme 3D (Left) and 2D schematic view of the binding (Right).

Metformin along with the natural ligand, and the inhibitor C30 furanone were docked into into the active site of the quorum sensing protein LasR. Metformin made four H-bonds with Asp73, Thr75, Thr115 and Ser129 and showed electrostatic interaction with Asp 73 (Fig. 11). The C30 furanone a known inhibitor to LasR was found to bind through H-bond with Trp60 and Arg 61 while the natural ligand makes H-bond with Tyr56, Trp60, Asp73 and Ser129 (Fig. 12). The MolDock scores for metformin, C30 furanone and the natural ligand were −82.37, −80.47 and −164.49, respectively. The inhibitor lacks the hydrophobic side chain that induces the correct formation of the hydrophobic core of LasR and this is the case in metformin.

**Figure 13:**
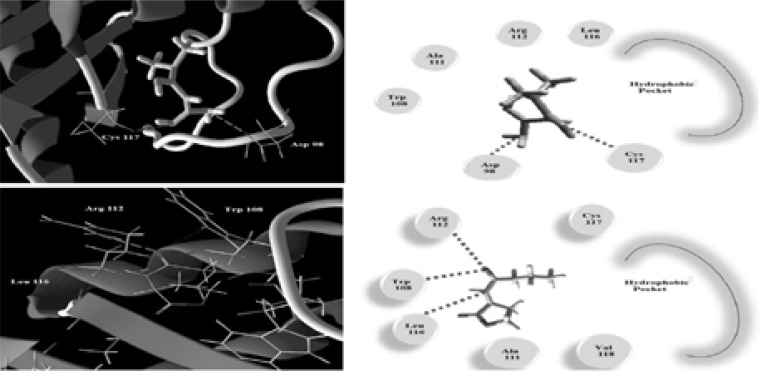
The Molecular docking of C4-HSL (Top) and metformin (Bottom) into the active site of rhlr receptor model 3D (Left) and 2D schematic view of the binding (Right).

Also, metformin was docked into rhlr receptor model active site. Its interaction to the receptor can be described by two H-bond with Cys117 and Asp98, while the auto-inducer C4-HSL makes H-bond with Leu112, Trp108 and Arg112 (Fig. 13). These interactions resulted in binding energy of −74.16 and −85.24 for metformin and C4-HSL respectively. The hydrophobic acyl group of C4-HSL is important factor for the conformational change to be an inducer that is absent in metformin which make it a possible inhibitor.

## Discussion

Quorum sensing represents a highly valuable target for modern anti-virulence approach that tends to bypass the problem of emergence of antibiotic resistance. This is because of its role in regulation of virulence factors production together with biofilm formation and bacterial motility^32^.

Inhibition of QS is usually investigated via the competitive inhibition of auto-inducers binding to their cognate receptors. However, most investigated quorum sensing inhibitors were toxic and cannot be used clinically^33^. To solve this problem, screening of FDA approved drugs for possible quorum sensing inhibition may be a useful alternative option that enables their clinical application for treatment of infections. This approach was found to be successful in our previous study on glyceryl trinitrate^34^. In the present study, the oral hypoglycemic drug metformin was tested for its potential quorum sensing inhibition. Metformin could inhibit PAO1 growth at 100 mg/ml. metformin (1/10 MIC) that showed no effect on bacterial viability was used to detect the effect of metformin against quorum sensing and virulence of PAO1.

*Chromobacterium violaceum* CV026 was used as a biosensor of quorum sensing. This mutant can produce the purple violacein pigment only when acylhomoserine lactones are added to the growth media. This pigment is produced under the control of CVi/R QS system^35^. Quantitative assay of violacein production was performed in the presence and absence of sub-inhibitory concentration of metformin, and it was found that metformin could reduce the pigment production. Furthermore, the possible interaction of metformin with LasR and rhlR receptors was investigated by molecular docking. Metformin could bind with LasR via hydrogen bonding and electrostatic attraction. The docking score of metformin was slightly lower than C30 furanone, so its binding ability is more or less similar to that of C30 furanone. Considering the interaction with rhlR receptor, the docking scores of metformin and the natural ligand butanoyl homoserine latone indicate comparable abilities of binding to rhlR. These results indicate that metformin is a quorum sensing inhibitor in PAO1.

The study was then expanded to investigate the activity of metformin against quorum sensing regulated virulence factors. Metformin significantly decreased the production of pyocyanin. Pyocyanin is responsible for production of reactive oxygen species by oxidation of reduced glutathione in the cell with concomitant reduction of oxygen^32^.

Biofilm formation and development is affected by quorum sensing^36^. Metformin, at sub-inhibitory concentration, showed a good biofilm inhibiting activity.

Quorum sensing is involved in resistance of PAO1 to oxidative stress due to interference with phagocytosis and intracellular killing by reactive oxygen species in neutrophils and the production of catalase and superoxide dismutase^37^,^38^. Metformin could increase the sensitivity of PAO1 to hydrogen peroxide.

Motility is linked to bacterial adhesion and subsequent biofilm formation. QS LasI/R and rhlI/R regulate bacterial motility^39^. Moreover, QS-deficient strains with impaired motilities can form thin and disperse biofilms^40^. Metformin could reduce both swimming and twitching motilities of PAO1.

In this study, metformin inhibited hemolysin, elastase and protease to different extents. The anti-hemolytic activity was higher. *Pseudomonas aeruginosa* produces these hydrolytic enzymes to enable the spread of bacteria inside the host tissues and resistance to the host immunity^41^.

## Conclusion

Metformin is a novel quorum sensing inhibitor in PAO1 that has the merit of being FDA approved for human use. It can be used as anti-virulence agent in the treatment of *P. aeruginosa* infection.
